# Modal Identification of Output-Only Systems of Composite Discs Using Zernike Modes and MAC

**DOI:** 10.3390/s19030660

**Published:** 2019-02-06

**Authors:** Minh Nguyen, Angelos Filippatos, Albert Langkamp, Maik Gude

**Affiliations:** Institute of Lightweight Engineering and Polymer Technology (ILK), Technische Universität Dresden, 01307 Dresden, Germany; minh.nguyen@tu-dresden.de (M.N.); albert.langkamp@tu-dresden.de (A.L.); maik.gude@tu-dresden.de (M.G.)

**Keywords:** modal assurance criterion, Zernike polynomials, operational modal analysis

## Abstract

The analysis of the structural dynamic behaviour of composite rotor–discs by a valid description of the eigenfrequencies and mode shapes can provide significant information for action-taking before a failure occurs. Specifically, vibration-based diagnostic methods, which are able to take into consideration the interdependencies and sequential changes of the modal properties could benefit from such an analysis. Here, on the example of composite rotors, a correlation method for experimentally determined mode shapes is developed. For this purpose the Zernike polynomials are used to enhance the identification of mode shapes. Furthermore, the modal assurance criterion (MAC) in combination with the frequency response criterion and a data processing approach are applied in order to characterize changing modal properties of composite rotors. In addition, the developed algorithms can be further extended in order to simplify the experimental evaluation of the complex dynamic behaviour of composite structures.

## 1. Introduction

An unknown or a false understanding and subsequent description of the structural dynamic behaviour of composite rotors can lead to severe misinterpretations and false decision making. Especially in damage identification methods such as vibration-based diagnostics, a false classification of the vibration response to a damage state can result in a catastrophic failure. The goal of the current investigation is to gain information and knowledge regarding changes of the structural dynamic behaviour of composite rotors by developing a correlation method that is able to automatically identify the eigenfrequencies and the resulting mode shapes. The identification of modal properties is always influenced by structural and environmental changes, which can result in unpredicted dynamic behaviour such as mode shifting, splitting and swapping and therefore affects the correlation of the investigated mode shapes. In this paper, glass fibre reinforced composite rotors are investigated to study the influences of changes in modal properties, which where measured by a laser vibrometer. An output-only approach is used to examine the measurement data by applying a processing method that mainly focuses on the modal properties and more specifically on the eigenfrequencies and their corresponding mode shapes. To characterize changing modal properties the ZERNIKE polynomials and the modal assurance criterion (MAC) in combination with the frequency response criterion are applied. This method has been developed primarily to be used with circular symmetric structures, which allows us to define assumptions by which certain mode shapes can be associated to each other or excluded. For example, symmetric rotor structures with isotropic or polar orthotropic material behaviour, which correspond to those of a circular membrane, show such features.

### 1.1. Aim and Outline of the Paper

The aim is to develop a method that correlates the state-dependent modal properties of composite rotors and in the case of a series of several sequential states provides a dynamic response sequence. Then, multiple algorithms that provide a robust approach to identify and correlate the eigenfrequencies and their corresponding mode shapes are implemented and evaluated. A subsequent experimental modal analysis captures the dynamic behaviour of composite rotor. This is followed by the determination of the dynamic vibration behaviour in case of material alterations. Here, a multi-stage test procedure is used in which measurements and load tests are carried out under increasing loading. The resulting stresses have been determined and analysed during previous investigations [1]. In these investigations the MAC is applied combined with an enhancement method that is based on the modal assurance criterion and its variants. Furthermore a post-processing method is developed based on frequency deviation. The developed approach can be used to ease simulation-based approaches that are used to model the damage evolution of fibre reinforced structures [2,3]. By this method, a non-monotonic change of the eigenfrequencies caused by extensive inter-fibre failure could be identified and respectively associated to their mode shapes [4,5].

### 1.2. State-of-the-Art

In the field of structural dynamics, it is often of importance to identify the mode shapes, extract them from the measured data and to correlate them correctly to mode shapes of different states, conditions, or numerically determined mode shapes [6]. Furthermore, there is no quantifiable measure which reflects the correlation of repeatedly taken identifications of eigenfrequencies and their corresponding mode shapes. Other investigations have shown that significant changes in eigenmodes, specifically in eigenfrequencies, can be observed, such as mode shifting and mode splitting [7]. Although, changes can also manifest in the formation of the mode shapes themselves, which can have a negative effect in their identification. On the other hand those deviations can also provide information about local material alterations of a structure [8,9]. In most of these cases the later described MAC or its variations are commonly used to perform mode shape correlations. Nowadays, the identification and correlation of eigenmodes is used in the design process or for structural health monitoring of machines, such as wind turbines [10,11].

#### 1.2.1. The Modal Assurance Criterion

Many approaches have been developed over the years in order to correlate mode shapes. The most commonly used in literature is the modal assurance criterion [6,9,12]. It is often used for the correlation of experimental and numerical determined mode shapes and in particular for the optimisation and verification of parametric models. The MAC is very similar to the autocorrelation and forms the scalar product between two normalized eigenvectors, which can be expressed as follows

(1) $$\mathrm{MAC}\;\left(\theta_{1},\theta_{2}\right)=\frac{|\theta_{1}^{H}\;\theta_{2}{|}^{2}}{\left(\theta_{1}^{H}\theta_{1}\right)\left(\theta_{2}^{H}\theta_{2}\right)}\;,$$

where $\theta_{1}$ and $\theta_{2}$ are two 1-D vectors of equal length. By normalizing these vectors the MAC is insensitive to different scaling factors. However, since an offset or phase shift of the signals cannot be detected, it is sensitive to vectors with a small number of elements, as well as to the angle between two vectors. If they are perpendicular to each other, the MAC treats them as zero MAC$(\theta_{1}⊥\theta_{2})=0$, and as one at an angle of zero between them MAC$(\theta_{1}∥\theta_{2})=1$. The MAC is therefore a measure that checks the linear dependency and is subject to several restrictions [6].

#### 1.2.2. Use of Correlation Methods in Modal Analysis

Modal analysis provides the dynamic behaviour of a structure in the form of modal parameters, so called modes, which are represented by eigenfrequencies, mode shapes, and modal damping. If several structural conditions are considered, it is also possible to identify vibration-based changes. Appropriate correlation methods can help to detect and quantify the accordance of modal properties. In order to successfully compare experimentally or numerically determined data sets and their mode shapes respectively, and be able to validate the corresponding simulation models, a robust modal identification of the modal properties is necessary.

The MAC calculates the scalar product, and thus the comparison of eigenvectors of different length is a difficulty. This problem often occurs when comparing experimentally and numerically determined mode shapes and depends on the number of degrees of freedom (DOFs), whereby numerical models usually consist of a higher amount of DOFs. Therefore, a systematic extraction or interpolation of relevant DOFs must be carried out. In [12] a method is described to ease the identification of mode shapes, allowing for a computer-aided and partially automated evaluation of measured data. Another challenge arises when comparing vectors of mode shapes from circular components that seem to be similar but are rotated. This corresponds to a shift of all values representing a phase shift and occurs more often with circular symmetric or multi-symmetric mode shapes.

To avoid these issues, various extensions of the MAC have been developed over the years. Another possibility results from the so-called modal tracking [13,14,15]. Examples of other correlation methods are for example the direct mode comparison (modal scale factor), comparison of degrees of freedom (coordinate modal assurance criterion), weighting of DOFs (scaled modal assurance criterion), square root deviation (modal assurance criterion square root) as well as comparison of subsets of modes (partial modal analysis criterion) [6].

### 1.3. Nomenclature for Circular Mode Shapes

**Figure 1:**
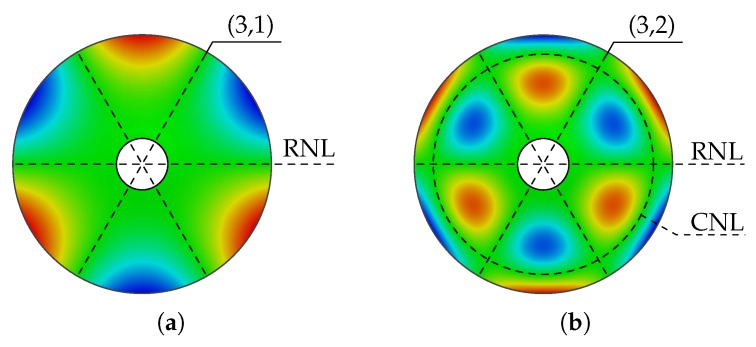
Illustration of the description rule for mode shapes of discs on two examples; mode shape (3,1) with only radial node lines (**a**) and (3,2) with radial and circular node lines (**b**).

To ease the understanding and the characterization of mode shapes, a description rule in the form of a nomenclature is introduced. The description is carried out by consideration of the established deflections perpendicular to a mode shape, based on [16]. Lines of radial nodes (RNL), also often called nodal diameters and circular nodes (CNL) can be used to introduce the following description rule,

(2) (n,m)withn,m∈N,

where *n* reflects the number of RNLs and *m* the number of CNLs. An example is given in Figure 1 and displays the two mode shapes (3,1) and (3,2) of polar orthotropic rotor–discs derived from numerical analysis.

Note that the inner circle is considered as a CNL due to the fact that the underlying structure has been fixed in the centre. The presented description provides a simple and understandable rule, that can also be applied to rectangular structures, whereby an edge has to be defined as the reference point (see Section 2.3 Step 1).

## 2. Underlying Theory of the Correlation Method

**Figure 2:**
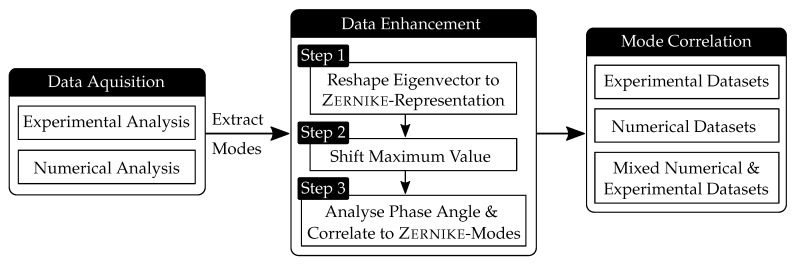
Flowchart of the data extraction and processing, showing the essential methods to improve mode shape correlation.

Local material fluctuations caused during the component production or by introduced damage can lead to strong differences in the formation of a mode shape. This issue can occur also with numerical models, which are based on a number of assumptions, e.g., a uniform material distribution. Experimental or numerical modal analyses can provide data sets for example in the frequency domain in the form of the power spectral density (PSD), from which the modes are extracted. The extraction of modes such as eigenfrequencies and mode shapes from PSDs is described in detail in Section 3. This section describes the implemented enhancement method and its required steps. In Figure 2 a flowchart is presented that illustrates the procedure used to improve mode shape correlation, which can be divided into 3 steps.

At the beginning the mode shapes are extracted from the examined PSDs. Subsequently the mode shapes are processed in three different steps that aim to improve the mode correlation, by reshaping the modes based on Zernike modes (**Step 1**), considering geometrical symmetry (**Step 2**) and the complex phase angle of the eigenvectors (**Step 3**). A method that associates mode shapes of changing conditions can be applied additionally and helps to reduce double modes. The method allows for the determination of frequencies that can be associated to valid eigenmodes or otherwise have to be considered invalid. The data enhancement includes several methods utilized to specifically improve the representation of the mode shapes. The data correlation can be performed between experimental or numerical data only, or in combination. In literature, the combined approach is often called mixed numerical experimental technique, which is used for example in the field of aerospace engineering [17]. The main focus of this paper is the mode shape enhancement to improve the results of the previously mentioned correlation methods. Each sub-function of the enhancement method is described in detail in the following sections.

### 2.1. Step 1: Abstraction of Mode Shapes Based on *Zernike* Modes

The single complex vectors that are extracted from separate PSDs are called the eigenvectors and are usually used to correlate mode shapes utilising the MAC. For composite structures such as disc-based rotors, however, it is reasonable to take a closer look at the symmetries and derive reduced mode shapes from them. The Zernike polynomials, which are orthogonal polynomials, are used to map wavefronts. The rotors investigated here exhibit mode shapes corresponding to the characteristics of such wavefronts. According to [18] Zernike polynomials are the product of the radius *r* dependent part $R_{n}^{m}\left(r\right)$ and the angle φ dependent part $G^{m}\left(\varphi \right)$,

(3) $$Z_{n}^{\pm m}\left(r,\varphi \right)=R_{n}^{m}\left(r\right)\cdot G^{m}\left(\varphi \right).$$

**Figure 3:**
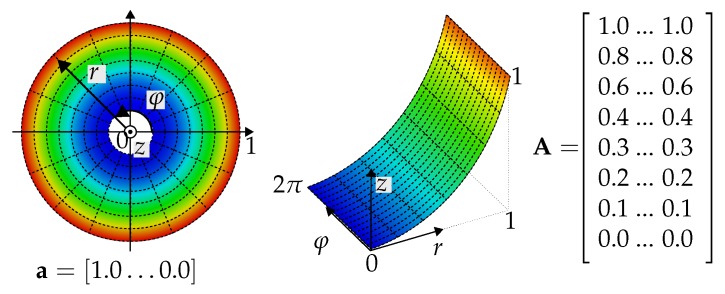
Decomposition of a proper mode shape into the two parts of the Zernike polynomial.

It is therefore possible to adequately describe each mode shape by using two polynomials or two interdependent mode representations. To determine these, the rotor is conceptually cut along an RNL and unfolds, resulting in a rectangular surface with the dimensions N×M, with N,M=[1,∞]. Following a path along one edge, for instance, along the radius, and meanwhile recording every vertical line, a vector of polynomials for every CNL can be created. Conversely, the vector of the RNLs is obtained by following a path along a CNL. In Figure 3, for instance, the fictitious vector a, containing real values in the range of [1.0,…,0.0], is reorganized according to the former described procedure, which leads to the matrix A.

Using suitable algorithms and mathematical functions to describe the RNLs and CNLs, the vectors are transformed into polynomials for *r* and φ. Thus, the Zernike decomposition is a function *f* which maps the eigenvector *u* of the length *N* to a matrix $U_{Z}$ with the dimensions n×m, where n,m are divisors of *N*;

(4) $$\begin{array}{cc} f:R^{N}\to R^{n\times m}:u\to U_{Z} & \mathrm{with}\;\;n\cdot m=N. \end{array}$$

The advantage of this representation is the possibility to perform operations across one dimension, thereby eliminating disturbances. For example, in cases where the changes of mode shapes themselves are negligible, averaging along a specific dimension can be used to reduce noise and determine mode shapes more accurately.

### 2.2. Step 2: Reordering of the Mode Shapes by Determining the Maximum Value

**Figure 4:**
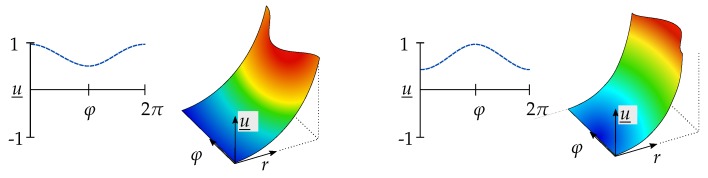
Mode shape (1,1) of a disc after decomposition into Zernike parts for two different extraction positions; the maximum value of the right mode shape exhibits a phase shift of π compared to the left.

During the extraction of the mode shapes from the PSDs, several difficulties can be encountered. For instance, the presentation of a mode shape along the node lines depends on the position at which the data extraction is initialized. Another common issue of determining mode shapes are the *moving mode shapes* or rotating mode shapes [7], which can be found more often in circular discs. These mode shapes can occur in experimental investigations where the RNLs appear not to be fixed to an angular position. Instead, the disc appears to rotate, similarly to the spinning of an Euler’s Disc [19]. The mode shape (1,1) after decomposition into fractions for two different extraction starting points is shown in Figure 4.

Without consideration of the rotational symmetry of circular discs u(φ=0)=u(φ=2π) apparently two different mode shapes can be observed, which in fact are the same mode shape with an offset of φ=π along the tangential direction. For the comparison of two mode shapes with the MAC, this offset must be corrected in advance. Therefore, it has to be defined that the maximum value is located in the RNL on the left edge and in the outer last CNL. For this purpose, the RNL which contains the highest value in terms of the magnitude is determined, and then all RNLs are shifted to the left and reinserted from the right. Note that absolute values have to be used, since there are positive and negative values. For further mentions, the described procedure is called maximum value shift (MVS) and its effect on the correlation can be observed in the example which is presented in Section 3.

### 2.3. Step 3: Examination of the Complex Phase Angle in Consideration of *Zernike* Modes

The measurement of structural conditions is always subject to external influences. Furthermore, the use of filters, window functions and averaging procedures can lead to errors that have an unintended effect on the transfer function; for example, frequency spectra can be averaged in absolute or complex terms. With absolute averaging the phase relation is lost and in this case the result is sometimes combined with the phase response of any spectrum. Since phase responses are subject to similar fluctuations as the frequency spectra themselves, there is a deviation in the transfer function. Thus, the amplitude and phase angle of a complex eigenvector are no longer in correct relation to each other. A further difficulty is the superposition of mode shapes, which arises e.g., through a combination of several successive modes caused by signal noise, signal interference, uneven excitation etc. Mode shapes with higher radial node numbers are especially more prone to be affected, since they do not necessarily have to be close to a mode shape with the same circular node number. Another issue can be closely spaced mode shapes were several mode shapes appear at nearly the same frequency. Since the appearance of this phenomena was marginal in the considered setup, solving this issue is not a goal of this work. Nevertheless, a suggestion is made in Section 2.6 describing a possible solution.

To differentiate the mode shapes and to compensate for the phase error it is necessary that the mode shapes are vectors whose elements are complex eigenvectors. These can be represented in the Euler form according to Equation (5) and form the product of a vibration amplitude $\hat{u}$ and Euler’s number with the complex phase angle iϕ in its exponent

(5) $$u\left(\phi \right)=\hat{u}\;e^{i\phi }\;.$$

The rotation of the phase angles of all eigenvectors of a mode shape can be considered as an oscillation, which is also often used for the animation of mode shapes. By rotating the angle it is possible that a mode shape becomes more noticeable, which allows for a differentiation in favour of the dominant mode shape. Mathematically the rotation of a complex vector represents a simple addition of the angles

(6) $$u^{*}\left(\phi^{*}\right)=\hat{u}\;e^{i\phi_{0}}\cdot e^{i\phi^{*}}=\hat{u}\;e^{i(\phi_{0}+\phi^{*})}\;\mathrm{with}\;\phi^{*}=\left[0,\frac{\pi }{2}\right]\;.$$

It should be noted that the damping part of damped vibration systems is included in the real part, and thus in highly damped systems the imaginary part can be neglected [20]. This allows us to limit the rotation to a range of [0,π], because further values represent the complex conjugate counterpart and are therefore repeating values, and the following applies

(7) $$\begin{array}{ccc} u^{*}\left(\phi^{*}=0\right) & =u^{*}\left(\phi^{*}=\pi \right)\;\mathrm{and}\;u^{*}\left(\phi^{*}=\frac{\pi }{2}\right) & =u^{*}\left(\phi^{*}=\frac{3}{2}\pi \right)\;. \end{array}$$

**Figure 5:**
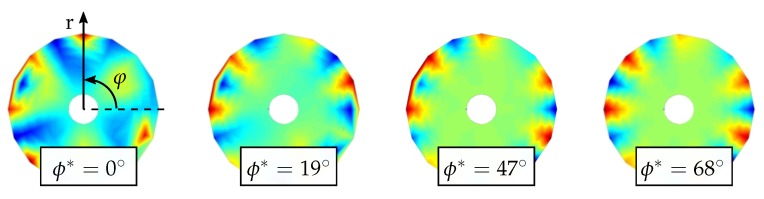
Affect of the phase angle on the representation of mode shapes on the example of the mode shape (7,1) at 4 different phase angles.

As an example, Figure 5 shows the mode shape (7,1) which is superimposed with the mode shape (3,2) in its initial state. The angle is represented in degrees (${}^{\circ }$) and rotated in 1${}^{\circ }$ intervals, which results in a total of 91 rotation steps.

In case of strong deviations in the averaging, the mode shapes cannot be determined properly. Using predefined mode shape matrices ensures high matching results only for actually possible mode shapes. For instance, the Zernike modes represent the totality of possible mode shapes of circular discs. Therefore, the mode shapes are correlated with predefined matrices of the Zernike modes. Note that only a fraction of modes have to be taken into account, since the mode shape order is restricted to half the number of sampling points of a mode shape.

### 2.4. Determination of Mode Shape Sequences Via Frequency Deviation Correlation

The condition-dependent dynamic behaviour of composite rotors can be determined based on the frequency shifts of the mode shapes. To evaluate the specific shifts a method is developed which identifies similar modes. More specifically, it determines sequential or successive paths from the MAC matrices and the associated frequencies, which represent the tendency of the frequency shift. Because some mode shapes can occur multiple times in the spectrum, the path with the lowest frequency deviation is determined using the nearest neighbour search algorithm [21]. Duplicate mode shapes can be identified using the auto-MAC (AMAC), which correlates all the mode shapes of one data set with itself, according to

(8) $${\mathrm{AMAC}}_{i}=\mathrm{MAC}\;\left(\theta_{i},\theta_{i}\right).$$

**Figure 6:**
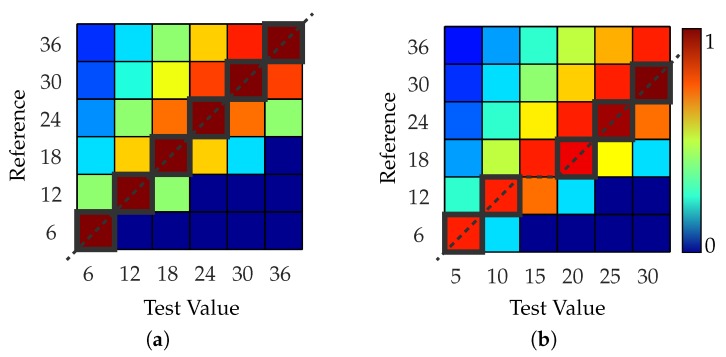
Frequency deviation correlation for (**a**) identical and (**b**) different pairs of input values and highlighted maximum correlation values in grey.

The resulting matrix is always diagonally symmetrical, whereby similar mode shapes lead to high correlation results off the diagonal. As an extension to the MAC, and to identify the frequency shifts, the frequency deviation correlation (FDC) is introduced, which determines the relative deviation of a frequency $\omega_{j}$ to a reference frequency $\omega_{ref,i}$ according to

(9) $${\mathrm{FDC}}_{ij}\left(\omega_{j},\omega_{ref,i}\right)=\left\{\begin{array}{cc} 1-\frac{\left|(\omega_{j}-\omega_{ref,i})\right|}{\omega_{ref,i}} & \mathrm{for}\;\omega_{j}\leq 2\;\omega_{ref,i}\;, \end{array}\right.$$

where $\omega_{j}$ and $\omega_{ref,i}$ represent the *i*-th and *j*-th eigenfrequency and ref represents the index of the reference frequency. To allow only values between [0,1] the restriction $\omega_{j}\leq 2\;\omega_{ref,i}$ is introduced, where 1 means that the frequencies are identical. In Figure 6 the FDC is shown for two vectors with identical values Figure 6a and different values Figure 6b.

The combination of AMAC and FDC allows for a more specific association of modal datasets. First, the deviation is determined separately, always between two following data sets and afterwards the mean value is calculated. A data set consists of the matrix of the frequencies and its associated mode shapes of an analysis step. A sequence consists of two consecutive analysis steps and contains the correlation of the two matrices and the frequency deviation of the respective mode shapes. The separate treatment is necessary, because when several similar mode shapes occur their tendency shift can be bigger than the difference between the nearest modes. Mode shapes which occur several times often show a phase shift, which is noticeable in a deviation of the MAC. The MAC value decreases with increasing phase shift and decreasing image quality. For each sequence $S_{i}$ the product (SP) is formed from the MAC and FDC of a path *P* and the mean value is calculated for each created path in the last step $S_{n}$:

(10) $$\begin{array}{c} {\mathrm{SP}}_{n}^{P}=\frac{1}{n}\sum_{i}^{n}{\mathrm{FDC}}_{i}^{P}\cdot {\mathrm{MAC}}_{i}^{P}\;\mathrm{with}\;i=1,2,\ldots n. \end{array}$$

**Figure 7:**
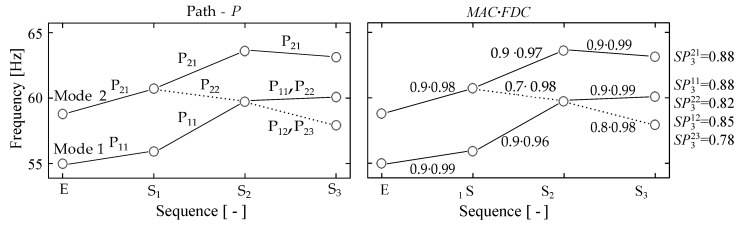
Paths derived from sequential determined frequency shifts of multiple modes (**left**) with associated modal assurance criterion (MAC) and frequency deviation correlation (FDC) values (**right**).

Paths with the same origin are then compared. Thereby those mode shapes can be sorted out by applying a threshold value for the SPs. If a mode shape occurs several times, the maximum value can be used to determine the mode shapes with the best correlation. The determined paths for two mode shapes to which the individual SPs have been assigned are shown in Figure 7, whereas *E* marks the initial measurement.

In Figure 7 the index *P* of SP consists of two digits. For computational purposes *P* is a simple consecutive number. For a better understanding the first digit of *P* in Figure 7 denotes its origin *E* and the second digit denotes into which parts the path has split. For instance, $SP_{3}^{21}$ originated from the path of Mode 2 and later split into $P_{21}$ and $P_{22}$ (see Figure 7 (left)), whereby the first part continues the initial path.

### 2.5. Assessment of Mode Shapes Considering Restrains and Symmetry Conditions

Restraining a structure causes local stiffening, thus resulting in frequency shifts and hinders the formation of mode shapes. Therefore, another method was implemented that also uses the Zernike modes to identify the restrained DOFs allowing for the approximation of the resulting displacement distribution. Since rotors are rotationally symmetrical structures, the transfer into matrix form by separating discs along one radius line results in symmetrical boundary conditions that are represented by the outer columns of the matrix. The amount of shifts is equal to twisting a mode shape by a specific angle around its central axis. For instance, shifting the columns of a matrix by half the total number of columns represents an angle twist of $\varphi =\frac{\pi }{2}$. Non-symmetric structures can exhibit mode shapes that appear to be similar but with values shifted along one dimension. Taking into account the restrains described above, further mode shapes can be excluded with a given symmetry of the structure. In addition, the progressions of possible mode shapes can be calculated under consideration of the sampling theorem [22] and the correlation between familiar and all possible mode shapes can be determined by the former described MAC.

### 2.6. Assessment of Closely Spaced Mode Shapes

Closely spaced mode shapes are mode shapes that appear to be present at the same frequency or at least within a very small range of frequencies, impeding their proper distinction. In this case, several modes may indeed be present and the method described in **Step 3** can be used to determine the probability of each mode, whereby the correlation to Zernike modes ensures the proper mode shape recognition. A threshold value can help to distinguish whether a mode should be considered valid or be omitted. In order to determine any mode shape different from Zernike modes, the formerly described AMAC can be used to correlate all the mode shapes that were gathered by stepwise rotating the phase angle. The values of the resulting MAC matrix will form blocks or groups respectively for several similar mode shapes, easing the selection of mode shapes.

## 3. Evaluation of the Method on the Example of Composite Disc–Rotors

**Figure 8:**
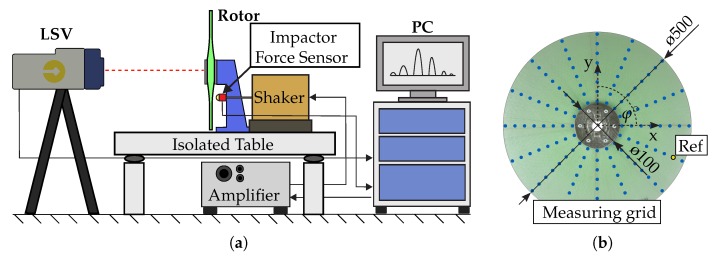
(**a**) Schematic set-up of the measuring equipment used in experimental modal analysis according to [5] and (**b**) rotor with measuring grid and dimensions.

To evaluate the developed approach, the correlation method was applied to experimental data gathered from glass fibre reinforced rotors using experimental modal analysis [23]. The investigated rotors consisted of preform and were fabricated using the tailored fibre placement (TFP) process [24]. The rotor geometry was based on previous investigations [5]. The dynamic behaviour of the rotors and the corresponding mode shapes was then investigated under a gradually increasing rotational load. In order to determine reference values for vibration-based damage analysis, the basic dynamic state of the rotors was investigated using the experimental set-up, as shown in Figure 8a. The data acquisition was performed by using a laser scanning vibrometer, an automated modal hammer and the software provided by the manufacturer of the laser. The investigation of the vibration response was carried out at 128 evenly spread measurement points as shown in Figure 8b.

The measured data has to be processed whereby the PSDs serve as the basis for modal identification. The selection of eigenfrequencies was performed in MATLAB by the function *Peakfinder*, to which a subsequent sampling was coupled. The selected peaks represent extreme values, that refer to the eigenfrequencies. On the basis of those values the relevant complex eigenvectors were read from the PSDs for each measuring point. Thus, vectors were created consisting of complex eigenvalues (Equation (5)). The procedure of this approach is also called peak-picking. It is one of the simplest methods to determine mode-shapes from PSDs [15]. In addition, a further deviation due to damage can be investigated, which can result in more distinctive changes in the dynamic behaviour, such as non-linear frequency deviations [5] and the formerly mentioned mode splitting.

### 3.1. Investigated Composite Rotors

Fibre reinforced materials exhibit a gradual damage behaviour which allows for extended operation of a structure even after initial damage has occurred [25]. The damage behaviour hereby strongly depends on the material properties and the structural design, regarding fibre and matrix material, reinforcement architecture, and geometry. Since the modal properties depend on the material properties, the use of modal analysis can provide knowledge of the condition of a structure [5,26].

**Figure 9:**
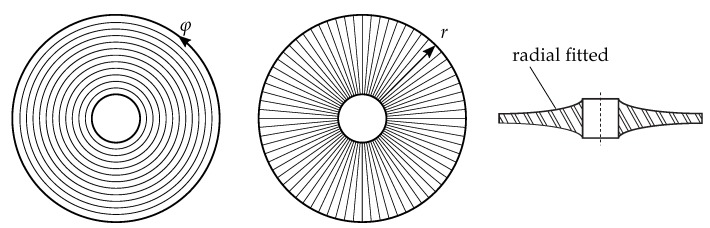
Fibre alignment of the two sub-preform types and rotor thickness profile.

The layers of the investigated rotors consisted of radially and tangentially arranged glass fibres, which were placed by means of tailored fibre placement. The preform consisted of several fibre layers and was produced from so-called textile sub preforms. The fibre orientation was radial (Ra) and tangential (Ta) and thus showed a polar orthotropic alignment, resulting in a planar, polar stress distribution along the radius *r* and circumference or angle φ respectively. The rotors featured a symmetric composite lay-up of Ra/Ta/Ra/Ra/Ta/Ra. The preforms were consolidated using the resin injection moulding process and a specifically designed mould that allowed for even material distribution, resulting in a double concave profile, decreasing in thickness from the inside to the outside as shown in Figure 9. In the following, results were evaluated to which the mode shape correlation of different measurements is of importance.

### 3.2. Identification of Eigenfrequencies and Extraction of Mode Shapes

**Figure 10:**
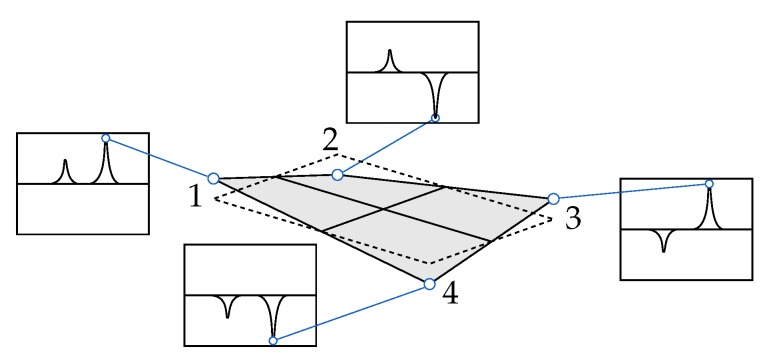
Display of a mode shape with associated parts of the power spectral densities (PSDs) for every extraction point.

The identification of eigenfrequencies was carried out by analysing the PSDs, which were provided by the experimental modal analysis. The frequency extraction was performed by the former mentioned peakfinder-algorithm, which offered a threshold value to handle signal noise. For instance, a low value may have resulted in a high amount of picked peaks, whereas a high value may not have produced any results. Subsequently, a selection of frequencies was carried out by sampling the values in a specified range. A smoothed regression curve was then formed for the region of interest to minimize noise. For instance, a noisy area will result in poor regression or a large residual, which leads to the exclusion of values. Once the frequencies were identified, eigenvectors for the given frequency range were extracted from all PSD lines. Each amplitude was a complex vector, which consisted of the magnitude and its relation to the other values, in the form of the complex phase angle. The resulting eigenvector represented the mode shapes in one-dimensional form. Each element of these vectors consisted of a complex vector and thus represented the second dimension of space. Through the reference to the measuring point, a three-dimensional mode shape was created. Using a rectangular plate as an example Figure 10 shows how the amplitudes are extracted from the PSD and then converted into an eigenvector or mode shape respectively.

It should be noted that the frequencies were determined from the averaged absolute values of the PSD. For a correct representation of the mode shapes however, it is necessary to extract the amplitudes in their complex form.

### 3.3. Evaluation of the Developed Approach

**Figure 11:**
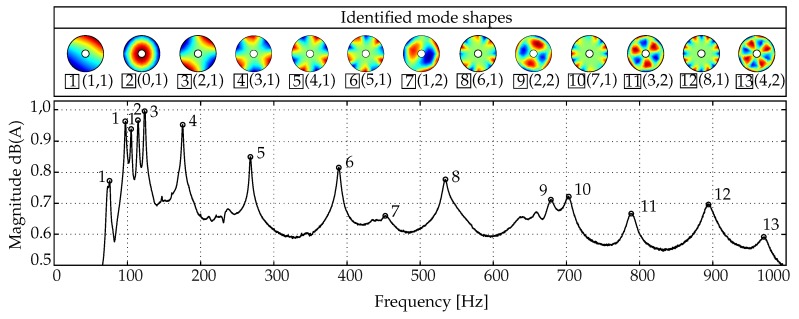
Display of mode shapes with associated frequency spectrum of a glass fibre rotor in its initial state.

To evaluate the efficiency of the previously described preparation and correlation methods, the gathered PSDs were analysed. An example of the frequency response and the extracted mode shapes of a rotor, determined in its initial and unaltered state, is shown in Figure 11. It can be noticed that only eigenfrequencies with clearly identifiable mode shapes were selected and furthermore, the notation, which is placed below each shape, was correctly determined.

**Figure 12:**
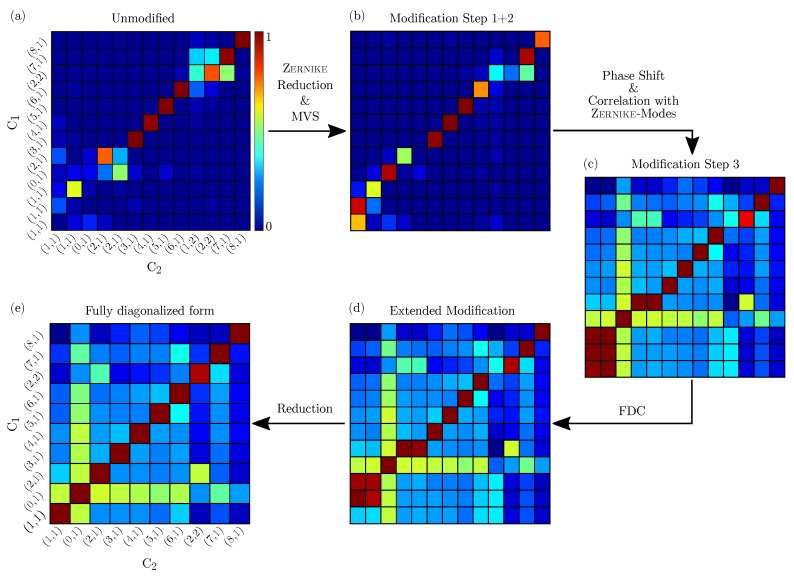
Several MAC matrices illustrate the correlation results of a rotor between two different conditions C${}_{1}$ and C${}_{2}$ and with different optimization methods applied, originating from unmodified eigenmodes (**a**), followed by enhancement methods such as Zernike reduction and maximum value shift (MVS) (**b**), phase angle modulation and direct correlation to Zernike modes (**c**), the extended correlation method FDC (**d**) and a final reduction steps (**e**).

Further on, the data sets consisting of the mode shapes were correlated against each other. Figure 12 shows an example that depicts the results of two individual correlation steps between consecutive load steps of a rotor in the form of MAC matrices. To alter the structural properties and thus cause changing modal properties, the rotor was exposed to a rotational speed-up test, as described in [5]. For all load steps there are several such matrices, from which the frequency shifts can be determined. The figure also illustrates the procedure, which consists of the following steps:

(*a*) Derive the MAC matrix from the unmodified mode shapes.
(*b*) Shift maximum values of the mode shapes and intersect result with (*a*).
(*c*) Derive the MAC matrix from the Zernike modes and intersect result with (*b*).
(*d*) Derive the FDC matrix from the eigenfrequencies and intersect result with (*c*).
(*e*) Sort out invalid and duplicate mode shapes.

The evaluation was always performed according to its purpose, e.g., if the frequency deviations were not significant, step (*d*) can be omitted, and directly passed to (*e*).

The first MAC matrix in Figure 12a represents the correlation result of the unmodified mode shapes and shows a much smaller number of matches than the MAC matrix in Figure 12e. Furthermore, 13 valid mode shapes could be identified for condition one (C1) and 12 for condition two (C2) resulting in a non-symmetric MAC matrix. For a stepwise examination, the correct mode shapes of both states had to be correlated. Shifting the maximum value improved the correlation of a few mode shapes, such as mode shape (1,1) and (0,1), which can be found in the lower left corner of (b). The correlation against Zernike modes, in contrast, created a much more destined result and was key in determining the mode shape notations. It should be noted that distinct shifts of the eigenfrequencies can be observed, which are dependent on certain types of mode shapes [27,28]. Those shifts formed blocks or flipped diagonals of several values, such as for the mode shapes (2,1) and (1,1) in (a). Another improvement became present from (b) to (c). For instance, compared to the first two correlation steps (a, b) the correlation values of specific mode shapes had increased, due to the now more evident similarities. Furthermore, in the lower left corner of (c) the multiple appearance of the mode shape (1,1) had formed a block of almost equal correlation values. This could be resolved by applying the FDC, which resulted in (d) and can be reduced to a single pair of modes by taking into account the angle of the initial mode shape orientation, the MAC values and the amplitude ratio of the corresponding spectra, leading to a reduced MAC matrix such as in (e).

## 4. Conclusions

For an improved investigation of the structural dynamic behaviour of polar-othotropic circular composite structures a new method was proposed and the capability to apply such methods to evaluate related changes in eigenmodes was investigated. Furthermore, experimental modal analysis were conducted to determine the quality of the developed methods. A satisfactory utilization of the method was found, offering the possibility to detect changing eigenmodes. This was enabled by applying an advanced correlation method, which uses Zernike polynomials and modes to enhance and identify the mode shapes on the example of glass fibre reinforced rotors. A stepped rotational run-up test of another investigation provided rotors with altered material properties, which were examined and allow for the determination of the condition-dependent vibration behaviour of composite structures. The methods can be used to improve correlation results of circular discs. Combined with the also described frequency deviation correlation, the sequentially changing condition of a structure can be examined and provide a better understanding of the underlying interdependencies.

Furthermore, closely spaced mode shapes should be considered in future investigations, because of its high importance in the area of identification of modal properties and its complexity regarding composite structures. A suggestion was made of how this issue may be resolved, but further research has to be carried out in order to evaluate its capability.

## Abbreviations

The following abbreviations are used in this manuscript:

MAC	Modal assurance criterion
FDC	Frequency deviation correlation
DOF	Degrees of freedom
MVS	Maximum value shift
PSD	Power spectral density
RNL	Radial node line/nodal diameter
CNL	Circular node line
